# ZPS: visualization of recent adaptive evolution of proteins

**DOI:** 10.1186/1471-2105-8-187

**Published:** 2007-06-07

**Authors:** Sujay Chattopadhyay, Daniel E Dykhuizen, Evgeni V Sokurenko

**Affiliations:** 1 Department of Microbiology, University of Washington, Seattle, WA 98195 USA; 2 Department of Biology, University of Louisville, Louisville, KY 40292 USA

## Abstract

**Background:**

Detection of adaptive amino acid changes in proteins under recent short-term selection is of great interest for researchers studying microevolutionary processes in microbial pathogens or any other biological species. However, independent occurrence of such point mutations within genetically diverse haplotypes makes it difficult to detect the selection footprint by using traditional molecular evolutionary analyses. The recently developed Zonal Phylogeny (ZP) has been shown to be a useful analytic tool for identifying the footprints of short-term positive selection. ZP separates protein-encoding genes into evolutionarily long-term (with silent diversity) and short-term (without silent diversity) categories, or zones, followed by statistical analysis to detect signs of positive selection in the short-term zone. However, successful broad application of ZP for analysis of large haplotype datasets requires automation of the relatively labor-intensive computational process.

**Results:**

Here we present Zonal Phylogeny Software (ZPS), an application that describes the distribution of single nucleotide polymorphisms (SNPs) of synonymous (silent) and non-synonymous (replacement) nature along branches of the DNA tree for any given protein-coding gene locus. Based on this information, ZPS separates the protein variant haplotypes with silent variability (Primary zone) from those that have recently evolved from the Primary zone variants by amino acid changes (External zone). Further comparative analysis of mutational hot-spot frequencies and haplotype diversity between the two zones allows determination of whether the External zone haplotypes emerged under positive selection.

**Conclusions:**

As a visualization tool, ZPS depicts the protein tree in a DNA tree, indicating the most parsimonious numbers of synonymous and non-synonymous changes along the branches of a maximum-likelihood based DNA tree, along with information on homoplasy, reversion and structural mutation hot-spots. Through zonal differentiation, ZPS allows detection of recent adaptive evolution via selection of advantageous structural mutations, even when the advantage conferred by such mutations is relatively short-term (as in the case of "source-sink" evolutionary dynamics, which may represent a major mode of virulence evolution in microbes).

## Background

Amino acid replacements in proteins may be advantageous in the course of an organism's adaptation to changing conditions in an established habitat or upon its spread into a novel habitat [1,2]. Such recently-acquired mutations may occur independently in genetically distinct allelic backgrounds, in small numbers per allele and in different protein regions. This makes it difficult to detect the signals of adaptive SNPs using traditional molecular evolutionary analyses, such as *K*_*a*_*/K*_*s *_(*D*_*N*_*/D*_*S*_) ratio [3], Tajima *D *[4] or Fu & Li D* [5] statistics, primarily due to an overwhelming level of pre-existing neutral SNPs (both synonymous and non-synonymous) in the loci under selection [6]. Additionally, the adaptive mutations may provide only short-term advantage to the organisms. This occurs in the course of so-called 'source-sink' dynamics of evolution, where species populations are continuously spreading from established, evolutionarily-stable reservoir habitats (sources) into novel, evolutionarily-untested habitats (sinks) that commonly are transient in nature [7]. In these cases, mutational adaptation to sink habitats may constitute a liability upon the collapse of sink habitat, due to functional trade-offs that these mutations generally demonstrate in the reservoir source habitat. The source-sink dynamic is characteristic, for example, of pathogenicity-adaptive (pathoadaptive) evolution of microbial pathogens [6,8].

We have recently developed Zonal Phylogeny (ZP) analysis, to detect adaptive amino acid changes in proteins under selection during short-term habitat adaptation [6]. Along each branch in a DNA tree, we indicate the number of synonymous and non-synonymous mutation information. Then, the synonymous-only branches are collapsed in the tree and the DNA tree is converted to a protein tree where each node corresponds to a evolutionarily unique structural variant. This minimizes the effect on the protein tree of nucleotide homoplasy and reversion events that obscure phylogenetic relationships of protein variants. ZP then separates structural variants of the protein into two categories, or zones: those encoded by multiple haplotypes (i.e., differing from each other by only synonymous SNPs) are assigned to the Primary zone, while each of the variants encoded by a single unique haplotype is assigned to the External zone. Accumulation of synonymous substitutions in genes that encode proteins from the Primary zone indicates their circulation over extended evolutionary time, thereby suggesting evolutionary stability of the protein variants. On the contrary, the External zone variants would have evolved relatively recently, because synonymous variation is yet to accumulate within the encoding genes.

The External zone variants are likely to be under positive rather than neutral or purifying selection (i.e. with mutations being of adaptive rather than of neutral or slightly deleterious nature) when: (i) their number is higher than expected relative to the frequency of Primary zone variants [6]; (ii) the amino acid replacements are more commonly occur in same positions (structural hot spots) [6]; (iii) silent SNPs along the connecting branches are relatively rare [6], and (iv) haplotype diversity (based on size and frequency of haplotypes) of the External zone is significantly higher than in neutrally-evolving genes [9]. Such statistical comparisons of the two zones show the unambiguous signature of positive selection in, for example, *fimH *and *papG-II *(encoding adhesin genes of mannose- and digalactose-specific fimbriae of uropathogenic strains of *Escherichia coli *respectively), but not in genes from the same strains that are involved in either fimbrial biogenesis or housekeeping functions [6,9].

Here, we present Zonal Phylogeny Software (ZPS) that computerizes ZP. ZPS uses DNA tree topology and haplotype alignment of a gene under analysis to recreate the DNA-based phylogeny, to demarcate the number of synonymous (or silent) and non-synonymous (or structural) changes along each branch, to separate haplotype nodes into Primary and External zones, and then to provide zone-wise information on amino acid substitutions, structural hot-spots and haplotype diversity.

## Implementation

The ZPS program presented here can be downloaded as zps.pl [see Additional file 1] to be run in command prompt under Windows environment. The attempt is, at one hand, to design a visualization tool to have insights onto a gene phylogeny based on distribution of synonymous vs. non-synonymous SNPs, and on the other hand, to incorporate quantitative statistical measures of recent adaptive evolution based on ZP analysis [9].

### Inputs

Two input files are used: (i) a DNA alignment in FASTA format (e.g., <*filename*> .*fasta*) [see Additional files 2 and 3] using a DNA alignment software, such as ClustalX [10]; and (ii) a maximum-likelihood DNA tree topology (e.g., <*filename*> .*ml.tre*) [see Additional files 4 and 5] generated by PAUP* [11]. In the representative haplotype name, the user should only use alphanumeric characters (i.e. only decimal digits and alphabets). To allow for haplotype size/frequency-based analysis, duplicate haplotypes need to be removed in the input files, but with the user marking haplotypes with multiple representatives in the dataset by *n*< no. of representatives> . For example, if *seqA*, *seqB *and *seqC *haplotypes are identical, the user should use *seqAn3 *(or *seqBn3 *or *seqCn3) *as input. If there is a single representative of a haplotype, the user can use the name as it is and the program would be able to detect it as '*n1*'.

### Outputs

There is one tree output – "zp_tree.dnd" where each node name (for example, 'E4-seqA-n3-2S/1N-A77D' or 'P3-seqE-n8-5S/0N') depicts (i) haplotype separation to either the External ('E') or Primary ('P') zone, with intermediate hypothetical (unresolved) nodes marked as 'H'; (ii) followed by an arbitrary number assigned to a protein variant encoded by the haplotype (e.g. 'E4' or 'P3'); (iii) original name of the representative haplotype and the user defined number of haplotypes that are identical to it in the dateset (e.g. 'seqA-n3' or 'seqE-n8'), with ZPS automatically adding '-n1' to the haplotypes with single representatives; (iv) number of synonymous(S)/non-synonymous(N) SNPs along the connecting branch (e.g. '2S/1N' or '5S/0N'), and (v) specification of amino acid changes due to the non-synonymous SNPs (e.g. 'A77D'). The ZPS output tree can be viewed with tree-presenting software, like TreeView [12] or HyperTree [13]. The latter application also enables usage of color coding to visually distinguish different type of haplotypes and branches. Keeping HyperTree in mind, ZPS generates an additional color-code file, for the output tree file, to color-code the Primary and the External zone representatives. Two color-codes have been used: blue for all the Primary zone haplotypes that exhibit same-protein silent variability and red for all the External zone representatives. To color-view "zp_tree.dnd" in HyperTree, the user needs to 'import colors' calling "color-zp_tree.txt" file.

There are two analytical outputs: "pairwise-variation.txt" and "analysis-results.txt". The former file details the positions and specific changes along each branch in the tree, while the latter presents (i) the Primary and External zone representatives; (ii) haplotype ratio (as a ratio of the number of External zone haplotypes to the total number of haplotypes in the dataset); (iii) position-wise structural mutation information, both overall and zone-based structural hot-spot frequency (as a ratio of the number of hot-spot structural mutations to the total number of structural mutations), and (iv) calculations of α and Simpson's diversity statistics [9].

## Results and Discussion

ZPS has been extensively tested with different genes from *Escherichia coli *of diverse origin [6,9,14,15], *Burkholderia cenocepacia *[16], *Vibrio vulnificus *and hepatitis C virus genotype 1 [unpublished data].

**Figure 1:**
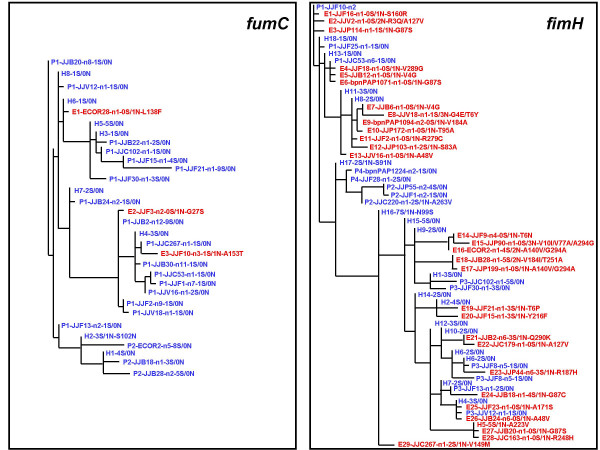
Comparative view of ZPS-generated trees for *fumC *and *fimH *genes of *E. coli *[9].

Figure 1 shows the color-coded outputs (using HyperTree) of the ZPS tree for two genes – *fumC *and *fimH *– of *E. coli *that encode housekeeping enzyme fumarase C and mannose-specific surface adhesin FimH. Even at first glance, one can see a relatively poorly developed External zone in *fumC *that suggests the presence of strong purifying selection (as expected for a housekeeping gene). At the same time, a massive External zone is quite evident in *fimH *that indicates relatively extensive recent evolution via amino acid changes.

The "analysis-results.txt" output includes the calculations to compare the patterns of evolution for different genes quantitatively, as shown in Table 1. The External zone frequencies of strains, haplotypes and structural hot-spots are significantly higher in *fimH *than in *fumC*. The diversity measures (Simpson's index, λ, and the α index value) show that the Primary zone λ and α values for the two genes are comparable (*p *> 0.50), suggesting the presence of long-circulated stable structural variants in the population of both FumC and FimH. The haplotype diversity of the Primary zone of *fimH *or *fumC *is significantly lower than the haplotype diversity of *fimH *External zone, but not of *fumC *External zone. In *fimH*, the low diversity of the Primary zone compared to the corresponding External zone could be hypothesized to be due to selective sweeps or bottleneck effects. However, the increased diversity of the *fimH *External zone can only be explained by positive selection, as we found its diversity being significantly higher than the diversity of both zones of *fumC *and, also, of Primary and External zones of three other genes from same strains – another housekeeping gene, *adk*, and type 1 fimbrial biogenesis genes, *fimI *and *fimC *[9]. At the same time, relatively high diversity was shown for External zone of *papG-II *gene encoding another, di-galactose-specific *E. coli *adhesin, indicating that adhesin genes could be prone to accumulation of adaptive amino acid changes under a short-term positive selection [9].

**Table 1:** Comparison of ZPS statistics for two genes: *fumC*, expected to be under strong purifying selection against structural variation as a housekeeping gene, and *fimH*, evolving under strong positive selection through SNPs as shown for genes encoding surface adhesins of pathogenic bacteria. The sample includes identical datasets of 75 strains for the two genes [9]. The *p*-values for the diversity measures are based on differential zonal haplotype diversity [9], while the other significance values are derived using 2 × 2 χ ^2 ^statistic. P and E denote Primary and External zones respectively

	zone	*fumC*	*fimH*	*p-values*
no. of strains	P	69	27	< 0.0001
	E	6	48	
no. of haplotypes	P	20	14	< 0.0001
	E	3	29	
zone-wise structural hot-spot frequency (no. of hot-spots/total no. of mutations)	P	0.00(0/1)	0.00(0/3)	1.00
	E	0.00 (0/3)	0.53 (19/36)	0.039
Simpson's index (λ)	P	0.11 ± 0.01	0.12 ± 0.03	0.002
	E	0.39 ± 0.10	0.07 ± 0.01	
α index	P	9.45 ± 1.80	11.71 ± 3.88	0.005
	E	2.39 ± 1.66	31.00 ± 8.25	

It is noteworthy that an advantage of ZP analysis of the haplotype diversity is that it considers both haplotype richness (i.e. total number of unique haplotypes) as well as frequency distribution (evenness) of these haplotypes in a zone. The latter feature of the diversity index incorporates the idea of relative fitness of a particular haplotype through the extent of its predominance in the sample set (provided the set is large enough, and relatively random).

To compare performance of ZPS with other commonly used methods for detecting signals of positive selection, we analyzed our datasets for *fumC *and *fimH *with codeml program implemented in the PAML package [17,18]. For each gene, we initially used two different models: one-ratio null model of neutral evolution (ω < 1) and one-ratio selection model of adaptive evolution (ω > 1). For *fumC *there is no difference (*p *= 1) between the log likelihood values of neutral (*lnL *= -1082.13) and selection (*lnL *= -1082.13) models. For *fimH *also, the neutral (*lnL *= -2245.44) and selection (*lnL *= -2243.58) log likelihood values are not statistically different (*p *= 0.16), though unlike *fumC*, the *p *value shows a possible trend toward selection. Thus, based on the entire tree, codeml was unable to detect unambiguously the presence of positive selection in *fimH*, demonstrating higher sensitivity of ZPS in this type of analysis. Then we used branch-specific selection model approach and assigned ω > 1 to clades containing multiple External zone nodes. For some of such clades on the *fimH *tree the log likelihood values for the selection model either differed significantly from the neutral model value (*p *< 0.0001), or differed considerably suggesting a distinct direction of selection (*p *< 0.11). No such difference was detected for the *fumC *clade that contained two External zone nodes (*p *= 0.84). Thus, clade-specific codeml analysis confirmed presence of positive selection for non-synonymous mutations in *fimH*, but not in *fumC*. However, unlike codeml, ZPS does not require any preliminary knowledge about the clade composition to detect the selection. At the same time, ZPS can be used in combination with codeml to ease singling out of the clades or branches on gene tree that were derived under positive selection.

## Conclusions

Synonymous mutations are generally considered to be selectively neutral and to accumulate randomly at a constant rate for a given gene. ZPS utilizes DNA trees to differentiate haplotypes that have evolved with accumulation of silent variations from those derived only through amino acid replacements, enabling visualization of adaptive structural variations that have recently emerged under positive selection. Information about the presence of mutational hot-spots and comparative zonal statistics on the size and frequency of various haplotypes provides insights into the adaptive evolution of genomic loci in any organism, from virus to human.

## Availability and requirements

**Project name: **Zonal Phylogeny Software (ZPS)

**Project home page: **

**Operating systems: **Windows

**Programming language: **Perl

**Other requirements: **ClustalsX, PAUP* and any tree-viewing software, e.g. TreeView or HyperTree

**License: **GPL 

## Abbreviations

ZP – Zonal Phylogeny

ZPS – Zonal Phylogeny Software

SNPs – Single Nucleotide Polymorphisms

## Authors' contributions

SC designed the software, implemented it and drafted the manuscript. DED contributed to the idea of the zonal phylogeny and helped to draft the manuscript. EVS conceptualized the zonal phylogeny, designed the software and wrote the manuscript. All authors read and approved the final manuscript.

## Supplementary Material

Additional File 1 The Perl program code for ZPS. Click here for file

Additional File 2 The FASTA alignment input files of *fumC *and *fimH *genes respectively Click here for file

Additional File 3 The FASTA alignment input files of *fumC *and *fimH *genes respectively Click here for file

Additional File 4 The PAUP*-output tree files of *fumC *and *fimH *genes respectively as other inputs for ZPS. Click here for file

Additional File 5 The PAUP*-output tree files of *fumC *and *fimH *genes respectively as other inputs for ZPS. Click here for file
